# A combination of low-temperature radiofrequency thermocoagulation and pulsed radiofrequency of the bilateral Gasserian ganglion for bilateral trigeminal neuralgia due to multiple sclerosis: a case report

**DOI:** 10.1186/s40981-025-00764-1

**Published:** 2025-01-13

**Authors:** Mihoko Tamura, Masayuki Nakagawa, Yoichiro Abe

**Affiliations:** Department of Pain Clinic, NTT Medical Center Tokyo, 5-9-22 Higashi-Gotanda, Shinagawa-Ku, Tokyo, 141-8625 Japan

**Keywords:** Bilateral trigeminal neuralgia, Multiple sclerosis, Gasserian ganglion block, Radiofrequency thermocoagulation, Pulsed radiofrequency

## Abstract

**Background:**

Bilateral trigeminal neuralgia secondary to multiple sclerosis is an extremely rare condition. When Gasserian ganglion block is performed, it is necessary to achieve reliable long-term analgesic effects while avoiding treatment-related complications.

**Case presentation:**

A 49-year-old male with multiple sclerosis exhibited persistent dull pain and paroxysmal electric shock-like pain in his bilateral maxillary molars and mandible. He was diagnosed with trigeminal neuralgia due to multiple sclerosis. Due to severe side effects, it was difficult to achieve adequate pain control with medication alone. By performing low-temperature radiofrequency thermocoagulation and pulsed radiofrequency of the Gasserian ganglion while monitoring masseter muscle contraction, a satisfactory and rapid analgesic effect was obtained without masticatory atonia.

**Conclusions:**

To the best of our knowledge, this is the first case of bilateral trigeminal neuralgia due to multiple sclerosis in which low-temperature radiofrequency thermocoagulation combined with pulsed radiofrequency was successfully performed for pain relief without masticatory atonia.

## Background

Trigeminal neuralgia (TN) secondary to multiple sclerosis (MS) is caused by focal demyelination in the root entry zone of the trigeminal nerve or in the spinal trigeminal complex at the pontine [1]. The incidence of bilateral TN is reported to be 4% [2] among cases of TN without MS and 11–30% [3] among cases of TN associated with MS. It indicates that TN due to MS is characterized by a high rate of bilateral TN. Carbamazepine is not as effective as in typical TN, and muscle weakness, gait disturbance, and dizziness have been reported in MS patients [4]. Medications for neuropathic pain, such as pregabalin, are also options, but the validation of efficacy is not sufficient [5]. Besides pharmacotherapy, there are other treatment modalities such as microvascular decompression (MVD), stereotactic radiosurgery, and percutaneous Gasserian ganglion lesions for TN due to MS [4]. Fraioli et al. recommend radiofrequency thermocoagulation (RF) of the Gasserian ganglion because of its certainty, rapidity, and low recurrence rate [6]. However, all of these methods are less effective when applied to TN due to MS than when performed for typical TN [7, 8]. Therefore, more careful judgment is required for the indication of these treatments.

## Case

The patient is a 49-year-old male. He noticed weakness in his left lower extremity 24 years ago, and after further evaluation, he was diagnosed with MS. 17 years ago, bilateral facial pain appeared, which improved with carbamazepine. He was treated with interferon-beta 1-b, steroid pulse therapy, immunoadsorption plasmapheresis, fingolimod hydrochloride, natalizumab (genetical recombination), glatiramer acetate, dimethyl fumarate, and siponimod fumaric acid for MS. He gradually developed paralysis of the left lower extremity and began using a motorized wheelchair.

**Fig. 1:**
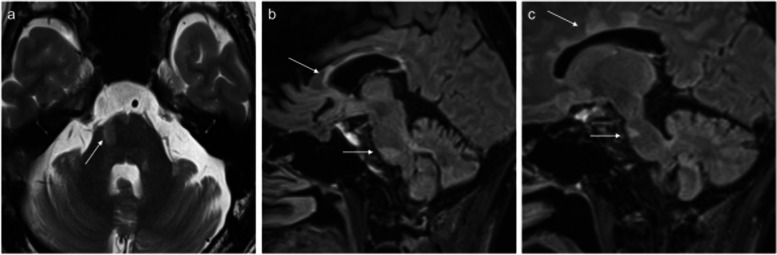
Brain magnetic resonance imaging. Fluid-attenuated inversion recovery images of brain magnetic resonance imaging showed high-signal areas (arrows) around the corpus callosum, lateral ventricles, and bilateral trigeminal nuclei in the bilateral pars ventralis pontis, suggesting demyelination. **a** Axial section, **b** right sagittal section, **c** left sagittal section

A year ago, bilateral facial pain flared up. Fluid-attenuated inversion recovery images of brain magnetic resonance imaging showed high-signal findings around the corpus callosum, lateral ventricles, and the bilateral trigeminal nuclei, suggesting demyelination (Fig. 1), which led to the diagnosis of TN due to MS. He was treated with steroid pulse therapy, but the effect was temporary. He was prescribed carbamazepine 700 mg/day, valproic acid 600 mg/day, tramadol 150 mg/day, acetaminophen 1300 mg/day, Neurotropin 4 N.U. (neurotropin units), duloxetine 20 mg/day, pregabalin 300 mg/day, and fentanyl patch 1 mg/day. However, due to severe side effects, it was difficult to achieve adequate pain control with medication alone. Because he had difficulty in eating due to pain, the performance of activities of daily living significantly deteriorated. He was referred to our department for interventional pain management. He had persistent dull pain (numerical rating scale: 7) in bilateral maxillary molars and mandible, and paroxysmal pain elicited by eating and talking. The pain was more intense on the left side and got worse at night. There was no facial allodynia or hypoesthesia.

**Fig. 2:**
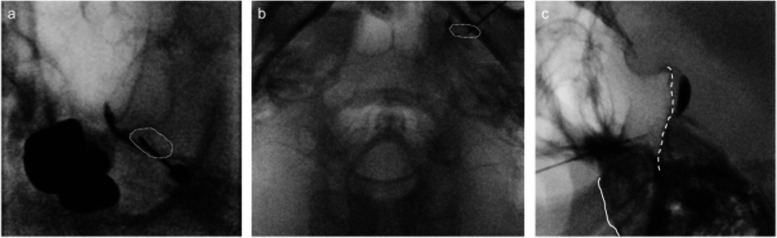
Left Gasserian ganglion block. The needle was inserted into the foramen ovale (dotted circle) in anterior–posterior oblique view. Contrast retention within Gasserian ganglion was confirmed. The direction of the needle was confirmed in the axial image, and the needle tip was confirmed not to exceed the clivus (dashed line) in the lateral image. Solid line indicates skull base. **a** Anterior–posterior oblique view, **b** axial view, **c** sagittal view

Left side Gasserian ganglion block (GGB) under fluoroscopy was performed (Fig. 2). TOP Lesion Generator (TLG-10; TOP, Japan) was used for RF. The foramen ovale was confirmed by anterior–posterior oblique view. A 22-gauge, 101-mm block needle with a 4-mm non-insulated tip (guiding needle KT; Hakko Medical, Japan) was inserted 3.5 cm lateral to the left corner of the mouth, following local anesthesia with 1% mepivacaine. Under sedation with propofol 50 mg, the needle was advanced into the foramen ovale until it reached the mandibular nerve. The motor component of the mandibular nerve was stimulated with the following parameters: output voltage 0.3 V, output frequency 2 Hz, and pulse width 1.0 ms. The needle was advanced until the contraction of the masseter muscle disappeared. Subsequently, we advanced the needle with the loss of resistance technique using iohexol, and confirmed its retention when the resistance disappeared. We confirmed the tip of the needle did not exceed the clivus in the lateral view. After injection of 0.2 ml of 2% mepivacaine, the entire region of the trigeminal nerve was anesthetized. Pulsed radiofrequency (PRF) was performed at 42 °C for 360 s, followed by RF at 70 °C for 120 s. The parameters of PRF were as follows: output voltage 45 V, pulse width 20 ms, pulse frequency 2 Hz, and output frequency 500 kHz. Left facial pain disappeared from the night of the day of treatment. The perception in the region of the second and third branch of the left trigeminal nerve was decreased to 7/10 and 5/10, respectively. Although right facial pain remained, he was able to discontinue pregabalin and reduce the daily doses of carbamazepine, tramadol, acetaminophen, and fentanyl patch from 700 to 500 mg, 150 to 100 mg, 1300 to 1000 mg, and 1 to 0.5 mg, respectively. The doses of other medications remained unchanged.

**Fig. 3:**
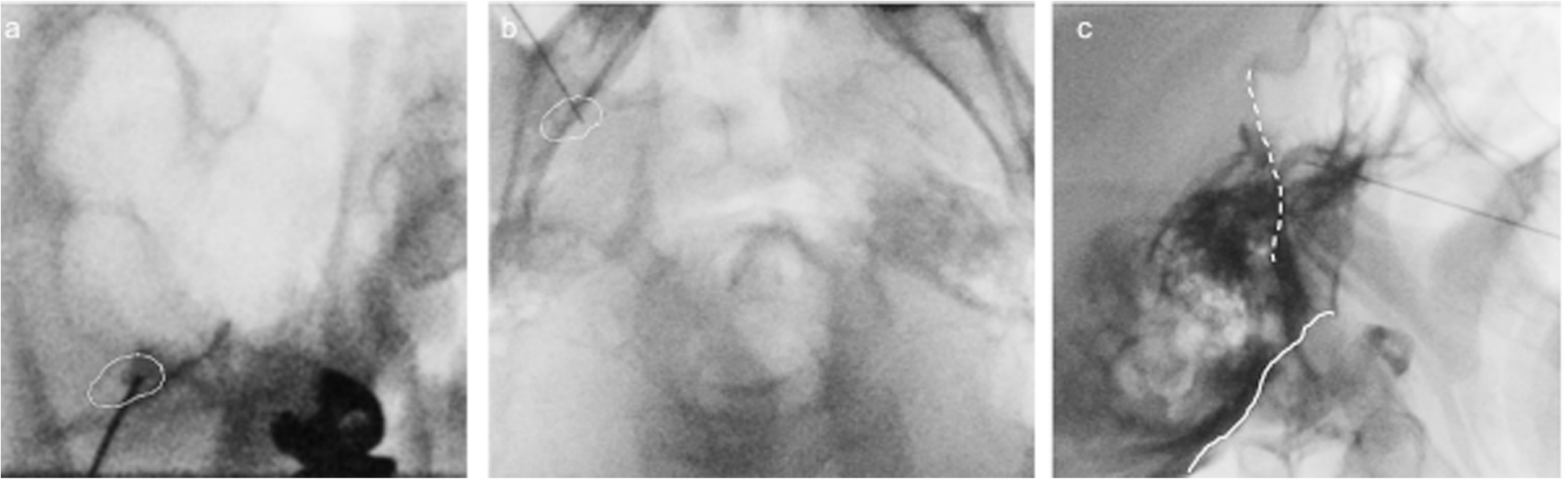
Right Gasserian ganglion block. The needle was inserted into the foramen ovale (dotted circle) in anterior–posterior oblique view. Contrast retention within Gasserian ganglion was confirmed. The direction of the needle was confirmed in the axial image, and the needle tip was confirmed not to exceed the clivus (dashed line) in the lateral image. Solid line indicates skull base. **a** Anterior–posterior oblique view, **b** axial view, **c** sagittal view

On the 116th day after left side GGB, pain in the right maxillary molar exacerbated. He exhibited persistent pain and paroxysmal pain elicited by shaving or talking. Right side GGB was performed using the same procedure as on the left side (Fig. 3). Immediately after treatment, right facial pain disappeared. The perception in the region of the second and third branch of the right trigeminal nerve was decreased to 7/10 and 5/10, respectively. As of now, there has been no recurrence of facial pain on both sides 464 days after left side GGB and 231 days after right side GGB. Although hypoesthesia remained (8–9/10 in the region of the second branch of the bilateral trigeminal nerve), there were no complaints of masseter muscle weakness. He was able to discontinue the fentanyl patch and reduce the daily dose of carbamazepine from 500 to 400 mg. The patient was highly satisfied with the treatment.

## Discussion

Secondary TN due to MS is a difficult condition that requires physician’s discretion to determine the appropriate treatment. In the present case, no vascular compression findings of the trigeminal nerve were evident; therefore, MVD was not indicated. Stereotactic radiosurgery, which requires 6–8 weeks to take effect [9, 10], was not suitable as an initial treatment because of the need for a rapid effect. RF of the Gasserian ganglion has less lethal complications, and effects can be obtained rapidly. The main complications of RF are hypoesthesia (96%), burning sensation (12%), masseter muscle weakness (4%), and deafferentation pain (about 0.8–4%) [5, 11, 12]. RF of the peripheral branches of the trigeminal nerve is an effective option to minimize treatment-related complications. However, we selected GGB because of the widespread distribution of pain and concerns about the burden placed on the patient by the need for frequent peripheral nerve blocks.

GGB using low-temperature RF combined with PRF is a novel neuromodulation method for TN [13–16]. PRF applies intermittent pulses of radiofrequency while keeping tissue temperature below 42 °C, and avoids permanent thermal damage. By inducing microscopic morphological changes in the nerve tissue, PRF provides long-term depression at primary afferent synapses [17], changes the expression of the pain-regulating C-fos gene [18, 19], induces antiallodynic effects [20], reduces neuroinflammation, and promotes tissue regeneration [21]. RF at temperatures below 70 °C combined with PRF is associated with fewer complications compared to temperatures of 70 °C or higher [22], although long-term analgesic effects have not been investigated. However, even if the procedure is associated with fewer complications, frequent recurrence and thus the need for repeated nerve blocks can be detrimental to patients. In the present case, because the need for contralateral GGB was anticipated in the future, it was necessary to ensure reliable long-term analgesic effects while avoiding masticatory atonia. PRF is also reported to promote recovery from complications caused by RF [23]. We therefore selected 70 °C, a temperature at which long-term analgesic effects have been reported [24], and combined PRF to promote recovery from any potential complications. We performed right side GGB after confirming that there was no masticatory atonia after left side GGB.

The risk of masticatory atonia caused by RF is reported to increase at temperatures of 65 °C or higher [25, 26]. Monitoring masseter muscle contraction can reduce the risk by distancing the electrode tip from the motor component of the mandibular nerve. This monitoring can be performed without any special equipment or complicated techniques. Hong et al. [25] recommend controlling the temperature of RF to ≤ 70 °C if muscle contraction is elicited at 0.1–0.3 V. The expansion of the lesion area generated by RF depends on the temperature and electrode dimension [27, 28]. The use of short non-insulated tips might prevent unnecessary tissue destruction.

Our case presented with paroxysmal electric shock-like pain which fulfilled the diagnostic criteria for TN. Meanwhile, he also exhibited characteristic symptoms of trigeminal neuropathy, such as nighttime pain and persistent pain. In a study comparing the efficacy of RF for typical TN against trigeminal neuropathy, the typical TN cases were shown to obtain superior prompt and long-term analgesic outcomes. One possible explanation for the favorable pain relief achieved in this case is that not only atypical but also typical symptoms of TN were presented bilaterally.

In a case of secondary bilateral TN, the indication for GGB should be carefully evaluated based on the mechanism, location of pain, and the patient’s general condition. When the bilateral GGB is performed, low-temperature RF combined with PRF and monitoring masseter muscle contraction may avoid severe complications and provide a highly satisfactory pain treatment.

## Data Availability

The data in this case report are available from the corresponding author on reasonable request.

## Abbreviations

GGB: Gasserian ganglion block
MS: Multiple sclerosis
MVD: Microvascular decompression
TN: Trigeminal neuralgia
PRF: Pulsed radiofrequency
RF: Radiofrequency thermocoagulation
